# Assessing the solubility, chemical stability and ecotoxicology of an emerging non-halogenated flame retardant, melamine cyanurate, against a prevalent halogenated congener, tetrabromophthalic anhydride

**DOI:** 10.1016/j.aquatox.2025.107537

**Published:** 2025-08-10

**Authors:** N. Masud, P. Hansal, B.D. Ward, J. Cable

**Affiliations:** a Cardiff University, School of Biosciences and Water Institute, Museum Avenue, CF10 3AX, UK; b Cardiff University, School of Chemistry and Water Institute, Park Place, CF10 3AT, UK

**Keywords:** Aquatic toxicity, Flame retardants, Melamine cyanurate, Dimethyl sulfoxide, *Daphnia magna*

## Abstract

Concerns over plastic-associated chemical toxicity are increasing amid the plastic pollution crisis. Halogenated flame retardants, though effective in plastic development, are being phased out due to toxicity, while nitrogen-based alternatives, such as melamine cyanurate (MC), are considered more chemically stable and less toxic. Here, we assess the solubility and chemical stability of MC in freshwater using various solvents and evaluate degradation after UV exposure. Additionally, we compare the acute and chronic aquatic toxicity of MC to the more widespread halogenated flame retardant tetrabromophthalic anhydride (TBA) using the *Daphnia magna* invertebrate model. Toxicity of a common solvent, dimethyl sulfoxide (DMSO), was also assessed. MC was insoluble in 16 of 18 tested solvents, with solubility only seen in a strong acid and base. UV exposure for 72 h within freshwater media indicated minimal degradation, classifying MC as a highly stable compound. Acute toxicity tests at 1–20 mg/L showed no significant difference in EC_50_ values between TBA (0.16 - 11.46 mg/L) and MC (5.91 - 13.23 mg/L). Chronic toxicity tests at 0.5, 5, and 15 mg/L yielded NOEC values of ≤5 mg/L for TBA and <5 mg/L for MC. At 15 mg/L, chronic exposure to TBA, MC, and DMSO resulted in 100% mortality in *D. magna*. These findings challenge the assumption that DMSO is a low-toxicity solvent in aquatic testing. Overall, the study highlights the difficulty in assessing the toxicity of highly stable flame retardants like MC, while indicating that they may exhibit similar aquatic toxicity as halogenated congeners.

## 1 Introduction

Common halogenated flame retardants such as TBA are typically found within unsaturated polyester and epoxy resins, plastics, paper, and textiles globally (Horrocks and Price 2001). They have become an essential component of thermoplastics, increasing their resistance to ignition and reducing the spread of fire. Some reactive flame retardants, such as TBA, also limit the amount of toxic gas produced when plastics burn (Ashford 1994; Parcheta-Szwindowska et al., 2024). However, concerns surrounding the safety of halogenated compounds, linked to their persistence, bioaccumulation and toxicity (PBT) have garnered a move towards developing safer compounds (Mitchel 2014; Jadhav 2018). Polychlorinated biphenyls (PCBs) and brominated flame retardants that can cause endocrine disruption and are potentially carcinogenic have been the major driver behind a move towards non-halogenated compounds (van der Veen and de Boer 2012; Liu et al. 2018). The broad categories of non-halogenated flame retardants are classed as organophosphates (e.g., triphenyl phosphate, diphenyl phosphate, tricresyl phosphate), inorganic compounds (e.g., aluminium trihydroxide, magnesium hydroxide) and nitrogen flame retardants (melamine cyanurate, melamine phosphates, dicyandiamide) (Morgan 2021). Nitrogen-based flame retardants have been gaining interest as they are advertised as of lower toxicity, with a preference for their solid-state form and the absence of dioxins and halogen acids produced when fires occur (Sangeetha et al. 2013; Jadhav 2018).

Despite some promising features of nitrogen-based flame retardants, their toxicity profiles remain poorly characterised. For example, MC, used in automotive, agriculture, construction, consumer goods and electronics industries, has virtually no associated ecotoxicological information (ECHA 2025a). In 2023, the global market for MC was valued at US $289.7 million, with projections indicating continued growth in the coming decade (Valuated Reports 2024). While melamine and cyanuric acid individually are considered to have low aquatic toxicity (Puschner et al. 2007; Reimschuessele et al. 2008; Pirarat et al. 2012), when combined to create MC it is potentially more toxic (Babayan and Aleksandryan 1985; Suchý et al. 2009). The solid and highly stable state of MC, attributed to multiple hydrogen bonds, may be a desirable trait by industry standards especially during handling and integration of flame retardants within plastic formulations. However, this inherent stability also makes it virtually insoluble in water and therefore challenging to test for aquatic toxicity. Paradoxically, this physical stability and insolubility may confer some degree of protection when aquatic exposure occurs due to the reduced chance of MC leaching out of the plastic (Luo et al. 2022). If, however, such leaching was to occur the insolubility may partition the flame retardant to an insoluble layer on the surface of aquatic bodies, leaving sedimentary and dietary consumption the primary means of exposure. For filter feeders, dietary consumption is likely to be the primary route of exposure for a highly stable and hydrophobic substance (OECD 2012, 2019).

In aquatic ecotoxicity studies where a substance of concern has been determined to be hydrophobic, the use of solvents to ensure homogenous exposure to the test substance is a standard procedure. While international standards encourage avoiding solvents with the acknowledgement that solvent toxicity is a potential concern, for certain hard to dissolve substances, their use is unavoidable (OECD 2019). Some of the most recommended solvents based on previous tests and OECD guidelines are acetone, ethanol, methanol, acetonitrile, dimethyl formamide and dimethyl sulfoxide (Hutchinson et al. 2006; OECD 2019). These recommendations are based on what solvents are considered of low aquatic toxicity and typically tested as a solvent control alongside negative controls to assess if the solvent is having toxic effects.

This study investigates the solubility, chemical stability and associated acute and chronic freshwater toxicity of the relatively novel non-halogenated flame retardant, MC, currently advertised as safer than halogenated congeners. This toxicity analysis is benchmarked against a prevalent halogenated flame retardant TBA. We also assess the acute and chronic freshwater toxicity of the common carrier solvent DMSO, that is widely considered of low aquatic toxicity and therefore used extensively within toxicity assessments (see ECHA 2003). To this extent, we used OECD Test No. 202 and 211 to assess acute immobility (48 h exposure) and chronic effects on reproduction (21 days exposure) of *Daphnia magna*, in addition to assessing heart and mandible rates of this freshwater invertebrate model.

## 2 Methods

### 2.1 Sourcing and synthesising flame retardants

TBA was sourced from Thermo Fisher Scientific (CAS: 632–79–1, purity >98 %). MC–CAS: 37,640–57–6- was synthesized at the Cardiff University School of Chemistry from melamine (CAS: 108–78–1, purity >99 %) and cyanuric acid (CAS: 108–80–5, purity >99 %), both sourced from Thermo Fisher Scientific. Details of melamine cyanurate synthesis are given in the material Supplementary Material (S1.1). For TBA, which has poor solubility in water, DMSO (CAS: 67–68–5, purity ≥99.9 %) was the solvent of choice to ensure homogenous exposure of *D. magna* and considered of low toxicity (OECD, 2019). The TBA stock solution 1 mg/mL was prepared by dissolving TBA in DMSO, heating to 100 °C and manually shaking from which all experimental concentrations were derived. This stock solution was kept in the dark at 20 °C. For MC, the choice of solvent was based on solubility tests (see 2.3 below) and information about any known aquatic toxicity profiles.

### 2.2 HPLC method development for dimethyl sulfoxide and tetrabromophthalic anhydride quantification

A high-performance liquid chromatography (HPLC) method was developed to simultaneously quantify DMSO and TBA in test solutions (Table 1). The method aimed to ensure accurate dosing of these chemicals in biological studies and to address challenges posed by TBA hydrolysing to its acid form in aqueous environments. TBA was fully hydrolysed in aqueous systems prior to analysis, and the HPLC method quantified the resulting tetrabromophthalic acid form. The HPLC system consisted of an Agilent 1200 equipped with a Zorbax column (15 cm × 4.6 mm, 5 µm) and a UV detector set at 210 nm. An isocratic mobile phase of 0.1 % phosphoric acid in a 50:50 acetonitrile-water mixture was used, with a flow rate of 0.8 mL/min and a total run time of 10 min. The method was validated for linearity, precision, and accuracy, with limits of detection (LOD) and quantification (LOQ) determined for both analytes. The LOD and LOQ were determined based on signal-to-noise ratios from the HPLC chromatograms. The LOD was defined as the concentration that produced a peak with a signal-to-noise ratio of 3:1, and the LOQ was defined as the concentration producing a signal-to-noise ratio of 10:1, in accordance with ICH Q2(R1) guidelines. This novel method enables simultaneous quantification of DMSO and TBA in a single isocratic HPLC run with UV detection at 210 nm. To our knowledge, no previous method has reported combined analysis of these two chemically distinct compounds under the same conditions. For details of HPLC standardisation and validity assessment see Supplementary Material S1.2.

### 2.3 Testing the solubility and chemical stability of melamine cyanurate

As MC is known to be virtually insoluble in water due to multiple hydrogen bonds giving an effectively infinite 2D sheet-like structure, and information on solubility in solvents is extremely poor, we tested the solubility of MC in 18 solvents (Table 2) covering all major solvent classes (Diorazio et al. 2016). For each solvent, 300 mg of MC was added to 75 mL in a flask, shaken for 2 min and left overnight before being filtered using a glass sinter grade 3 funnel. Samples were then dried in a 100 °C oven for 18 h and the resulting dry solid collected. Weight loss was measured to calculate the percentage of MC dissolved. All experiments were performed in triplicate, with water as a control due to MC’s known water insolubility (Sangeetha et al. 2013).

The stability of MC within aquatic environments after 72 h of UV exposure at 365 nm was then also tested. This stability was compared to MC exposed to UV without water. To this extent, samples containing 300 mg of MC were placed in glass vials either with 15 mL of distilled water or without any water and exposed to a UV lamp for 72 h. After UV exposure, the samples were filtered using a glass sinter grade 3 funnel, and the remaining solids were washed with distilled water and dried. For the dry samples that were exposed to UV, 15 mL of water was added, and the mixture was shaken for one minute. The percentage weight loss was calculated to assess the solubility and potential degradation of MC under these conditions. All experiments were performed in triplicate. To assess molecular mechanisms that may underpin potential MC degradation, due to UV exposure, a computational simulation was also performed. Specifically, density functional theory (DFT) calculations using the Gaussian 09 program with the PBE functional and def2-TZVP basis set were performed. Time-dependent DFT (TD-DFT) was employed to model the UV absorption spectrum and evaluate changes in MC’s electronic and geometric structure upon constant UV excitation (see Supplementary Material S1.6 for detailed explanation of DFT analysis). The aim was to determine whether UV exposure could disrupt MC’s planarity and hydrogen bonding network, which could explain if any mass change occurred in the UV exposure experiment highlighted above.

We also prepared potential water-soluble and UV degraded fractions of the MC, at 20 mg/L for subsequent *D. magna* exposure (see 2.5 below). The MC was sonicated in ultrapure reconstituted water for 72 h, with one stock solution exposed to UV under conditions highlighted above (Section 2.2) and the other protected from light by being wrapped in aluminium foil. All solutions were filtered through a grade 3 sinters to remove undissolved solids. Thus, the resulting filtrates would represent the potential water-soluble fractions from the UV exposed and UV protected MC for biological testing.

### 2.4 Test organism and culture conditions

*Daphnia magna*, obtained from ephippia bought from MicroBioTests in York (UK), were cultured at Cardiff University’s aquatics laboratory. *D. magna* cultures were maintained in 1 L glass aquaria (precleaned with detergent and ultrapure water) for four weeks from which new cultures were started with neonates (younger than 24 h and placed in precleaned 200 mL glass beakers. All cultures were maintained at 20±0.5 °C with a light-dark regime of 16L:8D h in reconstituted water (following OECD, 2004 standards) and the acute and chronic experiments detailed below were conducted under the same conditions. The aquaria were cleaned, and media renewed every two days. During this process, daphnids were carefully removed and returned by pipette (20 mL capacity with 5 mm opening). The daphnids were fed daily with a suspension of prepared synthetic food mix following ASTM recommendations (ASTM 2021). The reconstituted water, synthetic food and environmental conditions were consistent throughout the experiment. Determination of solid content in *Daphnia* water was required to ensure the *D. magna* food was providing adequate nutrition. As per the ASTM (2021) method, in brief: 10 mL of water containing the nutrients was added to a pre-weighed glass beaker and placed in a 100 °C oven for 24 h. Samples were placed in a desiccator before weighing. Samples were analysed in triplicate, and the weight difference was recorded. The solid content was between 1.7–1.9 g/L as per ASTM recommendations.

### 2.5 Acute toxicity test

To determine the acute toxicity of MC and TBA, *D. magna* individuals were exposed to these flame retardants in a 48-h immobility test, following OECD guidelines 202 (OECD 2004) to determine the effective concentration 50 (EC_50_). A stock solution of MC and TBA in the solvent DMSO at 1mg/mL was prepared, which was subsequently diluted into the 5 tested concentrations (1 mg/L, 5 mg/L, 10 mg/L, 15 mg/L, 20 mg/L-*n* = 5 daphnids per concentration) in 5 mL of reconstituted water. To ensure that *D. magna* survival was not being influenced by the potential water-soluble fraction of MC exposure, individuals were also exposed at the same five concentrations to reconstituted water sonicated and filtered with MC. The solvent DMSO, was also tested on its own in reconstituted water at the same 5 concentrations as the TBA and MC. A negative control treatment (i.e., without any flame retardant or DMSO solvent) was run alongside experimental treatments. Individual daphnids (<24 h old) were placed in the experimental and control treatments of 5 mL reconstituted water mixed with processed food (see above) in glass test tubes (single daphnids per 10 mL test tubes). After 24 and 48 h, we counted how many of the daphnids were immobilised, defined as completely unresponsive to gentle agitation of the test tubes.

### 2.6 Chronic toxicity test

To determine the chronic toxicity of MC and TBA, *D. magna* were exposed to flame retardants in 21-day reproduction tests following OECD guideline 211 (OECD 2012) for semi-static housing conditions. For each treatment (*n* = 20, <24 h old daphnids per treatment), pre-cleaned glass beakers were filled with 50 mL of reconstituted water and 200 μL of pre**-**prepared synthetic food. The MC and TBA stock solution (both in DMSO at a concentration of 1 mg/mL) were each tested at three concentrations (0.5 mg/L, 5 mg/L, 15 mg/L), along with a DMSO control and a negative control treatment (i.e., without any chemicals). The medium in each beaker for each treatment was renewed every 2 days. Each *D. magna* was fed 200 μL pre-prepared synthetic food daily. Throughout the 21 days, mortality and births were counted daily. Heart rate and mandible movements were taken every 7 days and on the same day water quality tests were performed. To measure heart and mandible rate, *D. magna* (*n* = 5) were randomly selected from each treatment and removed using a sterilised Pasteur pipette and placed in a glass petri dish within a droplet of reconstituted water. All *D. magna* were individually filmed with an iPhone 15 (Apple Inc.) attached to a laboratory clamp to film under Nikon SMZ800N microscope from 20 s video recordings (with a speed of 60 frames per second), heart and mandible rates were calculated per minute in QuickTime media player (Apple Inc.), a modified version of the methodology detailed in Sikorski (2021).

### 2.7 Statistical analysis

All statistical analysis was conducted in the R Studio (Version 2023.09.01). For GLM analysis of *D. magna* biological metrics (reproduction, mandible rate, heart rate and survival) in response to chronic exposure to MC, TBA, DMSO at 15 mg/L, 100 % mortality of *Daphnia* occurred before the first sampling on day 7 and therefore response variables for modelling were non-existent (i.e., because there were no organisms alive to perform a recording). Thus, no analysis for reproduction, heart and mandible rate are performed at 15 mg/L. For all models detailed below, underlying assumptions of models were assessed, i.e., normality of residuals, homogeneity of variance and identification of any outliers that may overly influence extracted coefficients from the models. For all models, Akaike Information Criterion (AIC) values were extracted in R Studio which measures goodness of fit for models, allowing model comparisons. Those with the lowest AIC value (i.e., lower AIC values indicating better model fit) were chosen (Thomas et al., 2013. For all modelling utilising generalised linear modelling (GLM), modifications were made in error families and link functions based on the nature of the data.

#### Solubility and chemical stability tests

to assess whether there was any significant difference in MC mass loss as a result of UV exposure and solvent tests using 18 major classes of solvents compared to water, a one-way analysis of variance (ANOVA) was performed where the response variable was the percentage mass loss.

#### Acute exposure

To derive EC_50_ values for acute 48 h MC and TBA exposure across five concentrations (1, 5, 10, 15 and 20 mg/L), we utilised a logistic regression model with a “cloglog” link function (i.e., allowing for more asymmetry in data distribution). For DMSO, EC_50_ values could not be meaningfully derived as no concentration led to 50 % mortality; the highest mortality was 40 %. Similarly for the water-soluble fraction of MC exposure treatments, there was 100 % survival in all treatments and therefore no survival analysis was performed.

#### Chronic exposure

For assessing how chronic exposure to MC, TBA and DMSO impacted total number of births (i.e., count data), a GLM with negative binomial error family and square root link function was utilised. For assessing how heart and mandible rate was associated with chronic exposure, a GLM with gaussian error family and log link function and Gamma error and log link function respectively were used. For the Kaplan-Meier parametric survival model, a Weibull hazard function was used to model survival across 21 days exposure with treatment at the three concentrations (1, 5 and 15 mg/L). To derive NOEC values for births within chronic exposure treatments, we utilised the coefficients from a GLM with negative binomial error family and log link function.

## 3 Results

### 3.1 HPLC dimethyl sulfoxide and tetrabromophthalic anhydride quantification

The method was validated for simultaneous quantification of DMSO and the hydrolysed form of TBA (tetrabromophthalic acid), which is the dominant species under aqueous conditions and validated for specificity, linearity, accuracy, precision, limit of detection (LOD), and limit of quantification (LOQ) to ensure reliable quantification of TBA and DMSO. The LOD and LOQ for TBA were 0.01 mg/L and 0.05 mg/L, respectively, while for DMSO, the LOD was 0.25 mg/L and the LOQ was 0.5 mg/L. At nominal concentrations of 1, 5, 10, 15, and 20 mg, the measured concentrations of TBA and DMSO are given in Table 1.

### 3.2 Testing the solubility and chemical stability of melamine cyanurate

The solubility screening experiment for MC demonstrated that MC remained insoluble in 16 out of 18 tested solvents, showing comparable results to water, which was used as a negative control (Table 2). There was no significant difference in solubility between these 16 solvents and water (*p* > 0.05 for all), indicating that MC does not dissolve appreciably in these solvent classes. Hydrochloric acid (37 %) and sodium hydroxide (10 M), however, showed significant solubilisation (*p* < 0.001, Table 2) with mean mass losses of 54.21 % and 19.24 %, respectively. These results suggest that while MC is highly resistant to dissolution under neutral and organic solvent conditions, it undergoes notable degradation in highly acidic and alkaline environments.

**Fig. 1:**
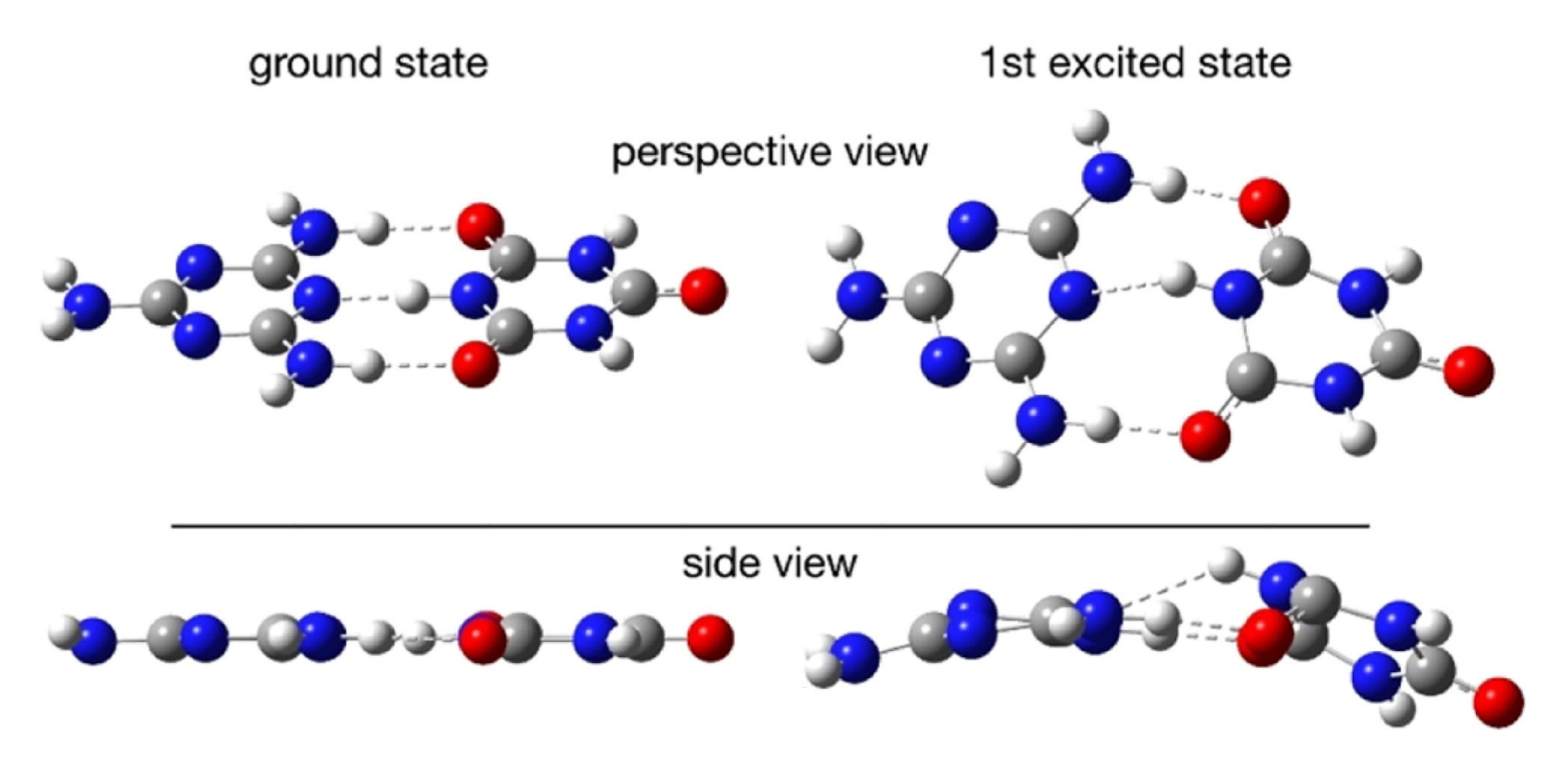
Molecular structures of a melamine cyanurate (MC) dimer obtained using density functional calculations. The ground state structures are on the left (i.e., not exposed to UV) whilst the first excited state on the right (exposed to UV). Both perspective and side views are provided which shows the deviation from planarity in the excited state.

MC exposed to UV light alone did not exhibit a significant mass loss when compared to the water control (*p* = 0.9982), indicating no significant degradation following UV exposure alone. However, when MC was exposed to UV in the presence of water, a significant 27 % mass loss was observed compared to the water control (*p* = 0.0040). Density functional (DFT) calculations were used to simulate possible changes to the molecular structure of MC under constant UV irradiation. If the excited state is sufficiently populated to allow geometrical changes to take effect rather than simply relaxing to the ground state, this could disrupt the hydrogen bonding network that holds the MC in an infinite sheet and could lead to a weakening of the structure, facilitating degradation pathways. The calculations did reveal that the excited state geometry deviates substantially from the planar structure found for the ground state not exposed to any UV (Fig. 1 and Supplementary Material S1.6 for detailed DFT analysis).

### 3.3 Acute exposure

**Fig. 2:**
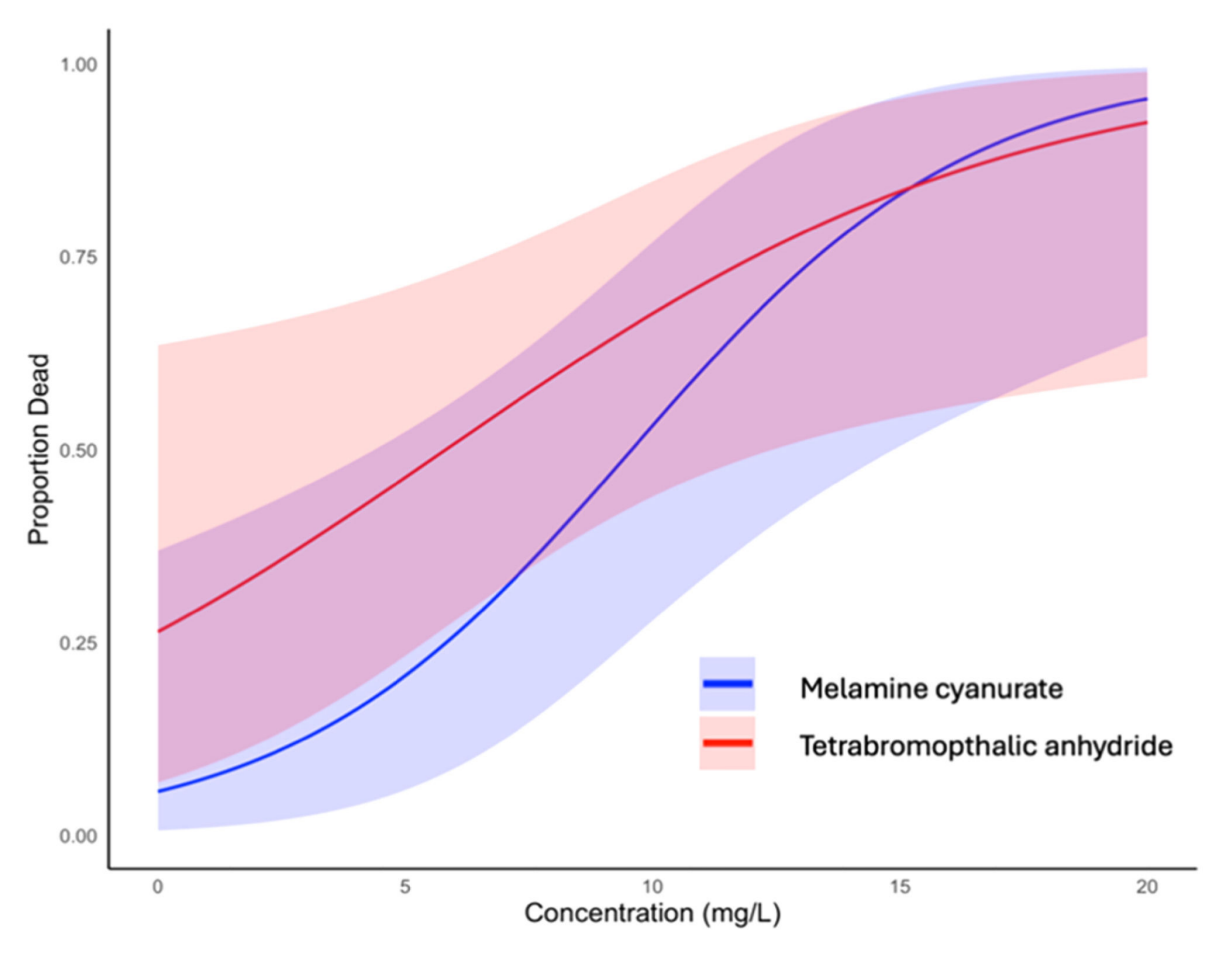
Prediction plots for effective concentration 50 (EC_50_) values based on acute toxicity, 48 h tests. This shows the probability of *Daphnia magna* death against concentrations of Tetrabromopthalic anhydride (TBA) and Melamine cyanurate (MC) extracted from the logistic regression model in R, showing the predicted values (lines) with 95 % confidence intervals represented as blue (MC) and red (TBA) shading.

The EC_50_ TBA range extracted from the binomial regression for TBA was between 0.16 - 11.46 mg/L which aligns with accepted values according to the ECHA classifications, and MC EC_50_ values were 5.91 - 13.23 mg/L (Fig. 2).

### 3.4 Chronic exposure

**Fig. 3:**
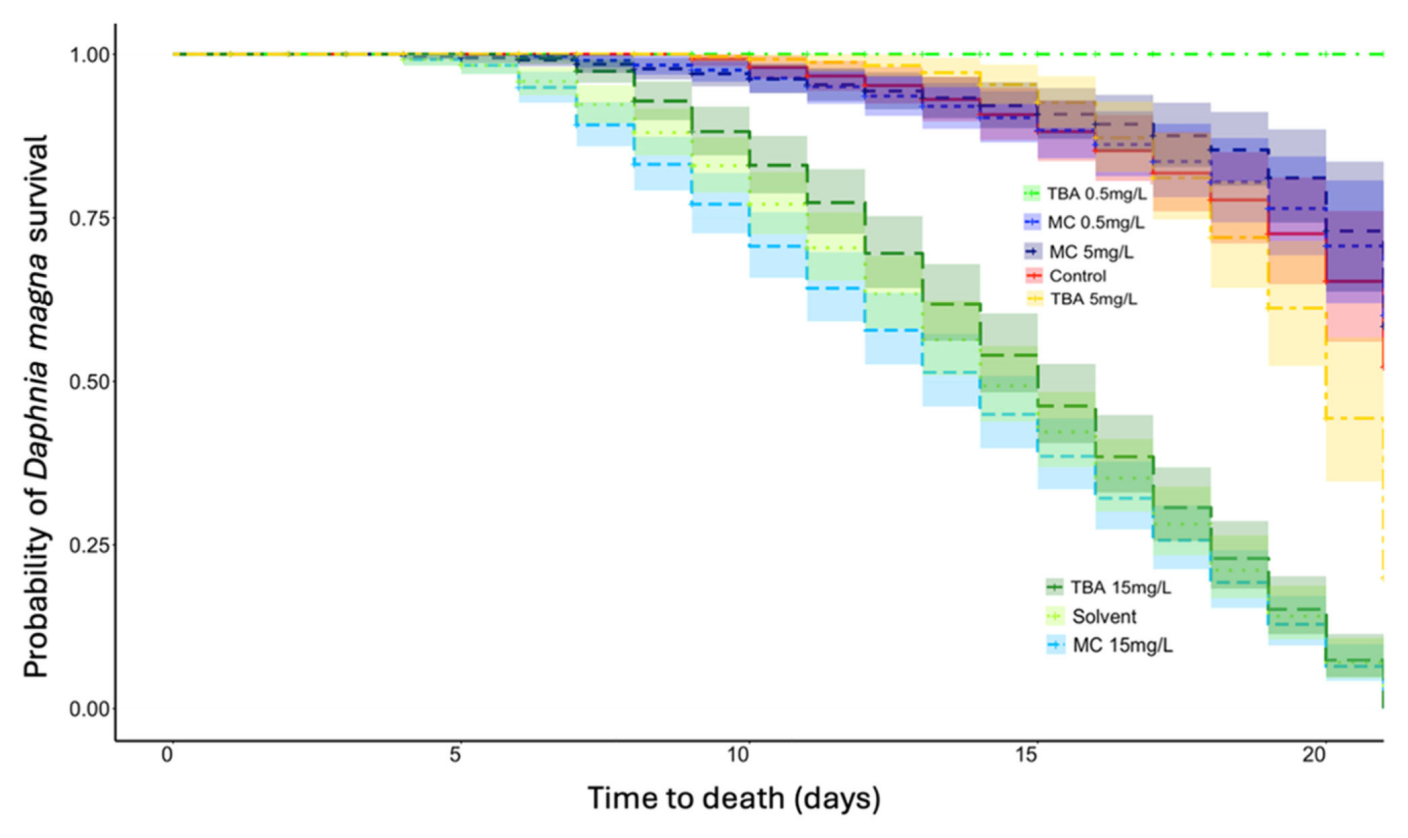
Survival probability line plots of *Daphnia magna* across 21 days of exposure to Melamine cyanurate (MC) and Tetrabromopthalic anhydride (TBA) at 0.5, 5 and 15 mg/L, with solvent (DMSO) tested at 15 mg/L, the concentrations used in all treatments, and negative controls also plotted with 95 % confidence intervals.

The Kaplan-Meier survival analysis with parametric modelling revealed that *D. magna* exposed to MC, TBA and the solvent DMSO at 15 mg/L had significantly lower survival compared to controls (*p* < 0.001 for the three treatments at 15 mg/L; Fig. 3). No significant difference was found between control survival (80 %) and MC and TBA at 0.5 mg/L (85 % and 100 % survival respectively) or 5 mg/L (80 % and 45 % survival) over 21 days exposure (*p* > 0.05). Importantly, unlike all the other treatments, MC and TBA but also the solvent DMSO at the highest concentration tested of 15 mg/L caused 100 % mortality within the 21-day exposure period.

**Fig. 4:**
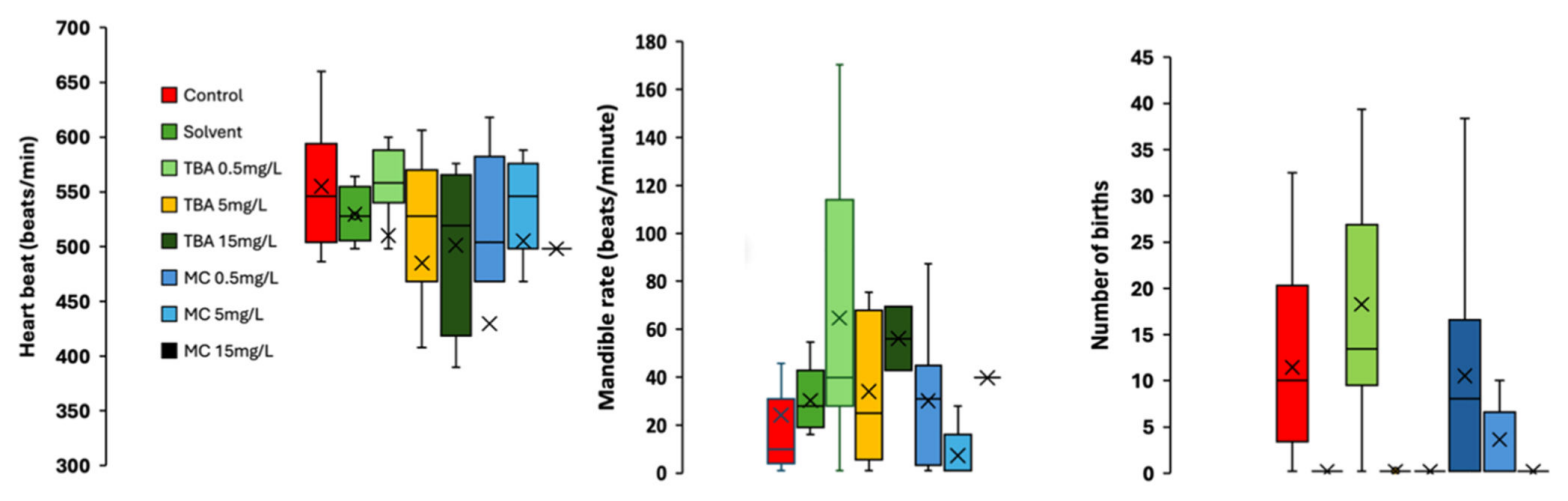
Boxplots of heart and mandible rate measured in beats per minute and number of births of *Daphnia magna* over 21 days of exposure to the solvent DMSO, and the halogenated and non-halogenated flame retardants tetrabromophthalic anhydride (TBA) and melamine cyanurate (MC). Shown is the median line, mean marker (x) interquartile range (box) and 1.5x interquartile range (whiskers).

The average births noted for the control treatment were 13 births per daphnid which, despite OECD and ASTM protocols implemented, falls below the recommended number for controls (see ASTM 2021 and OECD 2012). There were no births observed for *D. magna* exposed to DMSO, MC and TBA at 15 mg/L, as all died before maturity with none surviving after day 7. Nonetheless, significantly fewer births were noted for *D. magna* that were exposed to MC at 5 mg/L (*p* < 0.05) and no other significant differences were noted between controls and any other treatment at 0.5 and 5 mg/L (Fig. 4). Therefore, the NOEC for TBA and MC was ≤ 5mg/L and <5mg/L respectively. No significant differences were noted for heart and mandible rates between the controls and all other chronic exposure treatments across 21 days (*p* > 0.05; Fig. 4).

## 4 Discussion

### 4.1 Summary

In this study, solubility tests on the novel flame-retardant MC revealed that it was insoluble in 16 out of 18 tested major classes of solvents. Only strong acid and base solvents were able to achieve any dissolution for MC, both of which would have been extremely toxic for testing within aquatic systems and universally fatal for *D. magna*. Moreover, 72 h UV exposure at 365 nm in water caused 27 % degradation indicating the stability of the MC flame retardant. Ecotoxicity analysis of the legacy flame-retardant TBA and MC showed no significant differences in acute toxicity using the freshwater *D. magna* system, with the Effective Concentration 50 (EC_50_) values of 0.16 - 11.46 mg/L and 5.91 - 13.23 mg/L respectively for TBA and MC. Chronic toxicity analysis at 0.5, 5 and 15 mg/L revealed that the No Observed Effect Concentration (NOEC) were ≤ 5 mg/L and <5 mg/L respectively for TBA and MC. Perhaps most notable of all, this study challenges the widely held notion that DMSO, a widely used solvent in toxicity studies, is of low aquatic toxicity with this study showing that it was universally fatal for the *D. magna* tested at 15 mg/L, lower than previously reported NOEC values of 903.3 mg/L (ECHA 2003).

### 4.2 Nitrogen based flame retardant solubility, degradation and toxicity

The gradual phasing out and replacement of halogenated flame retardants with nitrogen-based alternatives is an increasing global trend (Sharkey et al., 2020). One such nitrogen-based flame retardant, is MC, that according to European chemical legislation, remains untested for its ecotoxicity (ECHA 2025). In the present study, MC proved to be very difficult to dissolve, with only strong hydrochloric acid and sodium hydroxide able to cause some dissolution. Furthermore, 27 % degradation of MC only occurred after 72 h continuous 365 nm UV exposure in aquatic media. UV exposure alone was not sufficient to cause any degradation, indicating that the combination of UV and water facilitates degradation. The Density Functional simulation-based calculations suggests that prolonged UV exposure could cause a decrease in the planarity of the MC molecule, which may enhance its susceptibility to further reactions, including potential interactions with water. These findings align with experimental data, which showed slight degradation of MC under combined UV and water exposure.

The fact that MC was virtually insoluble and demonstrated inherent chemical stability, presents a challenge for aquatic toxicity analysis as it is difficult to ensure homogenous exposure of the test chemical to the organisms. For filter feeders like *D. magna* OECD recommendations suggest using feed as the primary mode of dosing test organisms, which in the case of *D. magna* would typically be a liquid suspension of algae and yeast mix (OECD2019; Tkaczyk et al. 2021). Thus, the use of water would be unavoidable even when dosing with food for *D. magna*, which makes the hydrophobicity of MC an inherently challenging component in assessing toxicity within aquatic media. Moreover, due to the hydrophobicity of MC we could not use any standard analytical techniques to detect it within aquatic media (Sangeetha et al. 2013). Nonetheless, exposure to sonicated and filtered MC representing potential water-soluble fractions did not reveal any significant acute toxicity compared to control daphnids (i.e., there was 100 % survival).

The acute toxicity analysis of MC and TBA revealed that the EC_50_ values of both flame retardants overlapped significantly: 5.91 - 13.23 mg/L and 0.16 - 11.46 mg/L respectively, indicating that their toxicity profiles were not significantly different. This was based on the use of the solvent DMSO, to ensure as much homogenous exposure as possible but with the provisothat TBA is soluble and MC is not soluble in DMSO. The chronic toxicity, which measured life history traits fecundity, heartbeat and mandible rate (i.e., proxy for feeding), revealed that at 0.5, 5 mg/mL there were no significant differences noted for heart and mandible rates for any of the flame-retardant treatments. However, for fecundity, at 5 mg/L MC caused significantly fewer births compared to controls and the TBA treatment at the same concentration i.e., 5 mg/L. At 15 mg/L, MC, TBA and the solvent control for DMSO used to dissolve the flame-retardants were universally fatal with 100 % mortality achieved within 21 days exposure. It should be noted that the control daphnids in this study had fewer births than what is recommended by OECD and ASTM standards, and slightly lower than the solvent control daphnids and thus some caution is required when interpreting the results for fecundity. The dried mass of the food and water quality standards utilised for *D. magna* followed standard OECD and ASTM protocols and no signs of organism entrapment or pathogenesis were noted based on microscopic examination, so it is not clear why fecundity for the controls was low. The solvent DMSO is typically used as a water miscible carrier for the delivery of compounds to cultured cells and therefore it is possible that in the solvent control treatment, the DMSO was making nutrients more bioavailable to the daphnids, thus plausibly accounting for the slightly elevated fecundity (Summer et al., 2022).

More broadly, nitrogen-based flame retardants remain poorly studied for their toxicity. The other major melamine-based flame retardant, melamine polyphosphate also remains unclassified for toxicity by the ECHA (2025b) and therefore a read-across between shared functional groups of melamine polyphosphate and cyanurate is not possible to infer toxicity. There are nitrogen-based flame retardants such as cyanoguanidine, that differ structurally from MC, but are water soluble and have been shown to possess far lower aquatic toxicity compared to what was seen for MC in the current study, with an EC_50_ of 3177 mg/L (ECHA 1984), compared to 5.91 - 13.23 mg/L for MC.

Alongside the toxicity of the two flame retardants, we also tested the acute and chronic toxicity of the common solvent DMSO, commonly used in ecotoxicology to ensure homogenous exposure of test organisms to the test substance, and according to the European Chemical Agency its ‘..*toxicity profile for aquatic species is of low concern*’ (ECHA, 2003). Chronic exposure indicated that at 15 mg/L DMSO was universally fatal for *D. magna*, which varies significantly from effect concentrations stated by ECHA as 903.3 mg/L (ECHA 2003) for *Daphnia*; the value being derived by a (Q)SAR model. This does highlight the need to apply ecotoxicology values generated by structure-activity relationship models with caution.

### 4.3 Conclusion

This study investigated the solubility, chemical stability and fresh-water toxicity of an emerging non-halogenated nitrogen-based flame retardant, MC, benchmarked against a traditional halogenated flame retardant, TBA. The MC proved to be extremely difficult to dissolve in 16 of the 18 tested major classes of solvent, with only a strong acid and base able to cause some degree of dissolution. UV exposure at 365 nm for 72 h in aquatic media also caused some degradation in the molecular structure of MC, highlighting the robust molecular integrity of this flame retardant. Acute and chronic freshwater toxicity analysis with the *Daphnia magna* system revealed that the Effective Concentration 50 (EC_50_) values for MC and TBA were in the range 5.91 - 13.23 mg/L and 0.16 - 11.46 mg/L respectively. Chronic toxicity analysis at 0.5, 5 and 15 mg/L revealed that the No Observed Effect Concentration (NOEC) were calculated as ≤ 5 mg/L and <5 mg/L respectively for TBA and MC. At 15 mg/L chronic exposure of TBA, MC and DMSO was universally fatal (100 % mortality) for the *D. magna* population. Noting the challenge of testing the toxicity of a difficult to dissolve and highly stable MC, we cautiously report that the toxicity of MC is equivalent to the more traditional halogenated congener TBA. Notwithstanding the challenge of testing highly stable compounds under aquatic conditions, we suggest that future work focus on using structural analysis relationships, like QSAR to identify similar compounds with shared functional groups to infer potential toxicity. Moreover, this study reemphasises that single additive substitutions cannot be a sustainable solution in tackling plastic pollution.

## Figures and Tables

**Table 1:** TBA and DMSO concentrations used in the current study with ± standard deviations (SD) as measured by HPLC.

Compound	Nominal Concentration (mg/L)	Measured Concentration (mg/L) ± SD
TBA	1	0.7 ± 0.05
TBA	5	2.4 ± 0.14
TBA	10	5 ± 0.11
TBA	15	7.7 ± 0.12
TBA	20	9.6 ± 0.52
DMSO	1	2.2 ± 0.9
DMSO	5	3.0 ± 1.5
DMSO	10	5.6 ± 2.6
DMSO	15	18.2 ± 8.6
DMSO	20	26.2 ± 12.4

**Table 2:** Solubility screening results for melamine cyanurate (MC) in various solvents, with mass loss expressed as a percentage. Statistical significance was determined by using a one-way ANOVA against water*(MC is known as insoluble in water) as the control. Solvents with *p* > 0.05 were classified as insoluble, while those with *p* < 0.05 were classified as dissolved.

Solvent Class	Solvent	Mean % Mass Loss	Sth. Deviation	*P* value (compared to water)	Classification
Polar Aprotic	DMF	1.4	0.9	>0.9999	Insoluble
	DMSO	0.9	0.3	0.9999	Insoluble
Polar Protic	Water	1.3	0.6	N/A	Insoluble*
Polar Protic, Alcohol	Ethanol	0.8	0.1	0.9998	Insoluble
	Methanol	1.7	1.0	0.9999	Insoluble
Polar Aprotic, Ketone	Acetone	1.1	0.4	>0.9999	Insoluble
Halogenated	Chloroform	0.5	0.5	0.9997	Insoluble
	Dichloromethane	0.7	0.3	0.9997	Insoluble
Nonpolar	Cyclohexane	0.9	0.8	0.9999	Insoluble
	Hexane	1.6	0.9	0.9999	Insoluble
Ether	Diethyl Ether	1.6	1.3	0.9999	Insoluble
	Tetrahydrofuran (THF)	1.4	1.0	>0.9999	Insoluble
Ester	Ethyl Acetate	1.0	0.0	0.9999	Insoluble
Acid	Hydrochloric acid (12 M)	54.2	12.1	<0.0001	Dissolved
Alcohol	Isopropanol	1.6	1.2	>0.9999	Insoluble
Alkali	Sodium hydroxide (10 M)	19.2	12.2	<0.0001	Dissolved
Aromatic	Toluene	1.0	0.5	0.9999	Insoluble
	Xylene	1.0	0.4	0.9999	Insoluble

## Data Availability

All *Daphnia magna* biometric data will be made available via Mendeley.

## Data availability

Data will be made available on request.
